# Increased miR-155 in Microglial Exosomes Following Heat Stress Accelerates Neuronal Autophagy *via* Their Transfer Into Neurons

**DOI:** 10.3389/fncel.2022.865568

**Published:** 2022-05-11

**Authors:** Ping Li, Xue Luo, Zhen Luo, Gen-Lin He, Ting-Ting Shen, Xue-Ting Yu, Ze-Ze Wang, Yu-Long Tan, Xiao-Qian Liu, Xue-Sen Yang

**Affiliations:** ^1^Department of Tropical Medicine, Army Medical University, Chongqing, China; ^2^Key Laboratory of Extreme Environmental Medicine, Ministry of Education of China, Army Medical University, Chongqing, China

**Keywords:** heat stress, exosome, microglia, neuronal autophagy, miR-155

## Abstract

**Background:**

Heat stroke is the outcome of excessive heat stress, which results in core temperatures exceeding 40°C accompanied by a series of complications. The brain is particularly vulnerable to damage from heat stress. In our previous studies, both activated microglia and increased neuronal autophagy were found in the cortices of mice with heat stroke. However, whether activated microglia can accelerate neuronal autophagy under heat stress conditions is still unknown. In this study, we aimed to investigate the underlying mechanism that caused neuronal autophagy upregulation in heat stroke from the perspective of exosome-mediated intercellular communication.

**Methods:**

In this study, BV2 and N2a cells were used instead of microglia and neurons, respectively. Exosomes were extracted from BV2 culture supernatants by ultracentrifugation and then characterized *via* transmission electron microscopy, nanoparticle tracking analysis and Western blotting. N2a cells pretreated with/without miR-155 inhibitor were cocultured with microglial exosomes that were treated with/without heat stress or miR-155 overexpression and subsequently subjected to heat stress treatment. Autophagy in N2a cells was assessed by detecting autophagosomes and autophagy-related proteins through transmission electron microscopy, immunofluorescence, and Western blotting. The expression of miR-155 in BV2 and BV2 exosomes and N2a cells was measured using real-time reverse transcription polymerase chain reaction. Target binding analysis was verified *via* a dual-luciferase reporter assay.

**Results:**

N2a autophagy moderately increased in response to heat stress and accelerated by BV2 cells through transferring exosomes to neurons. Furthermore, we found that neuronal autophagy was positively correlated with the content of miR-155 in microglial exosomes. Inhibition of miR-155 partly abolished autophagy in N2a cells, which was increased by coculture with miR-155-upregulated exosomes. Mechanistic analysis confirmed that Rheb is a functional target of miR-155 and that microglial exosomal miR-155 accelerated heat stress-induced neuronal autophagy mainly by regulating the Rheb-mTOR signaling pathway.

**Conclusion:**

Increased miR-155 in microglial exosomes after heat stroke can induce neuronal autophagy *via* their transfer into neurons. miR-155 exerted these effects by targeting Rheb, thus inhibiting the activity of mTOR signaling. Therefore, miR-155 could be a promising target for interventions of neuronal autophagy after heat stroke.

## Introduction

Heat stroke is a medical emergency characterized by severe hyperthermia and multiorgan dysfunction, predominantly encephalopathy. Once onset, it progresses rapidly, and the fatality rate can be as high as 60% (Argaud et al., 2007; Hausfater et al., 2010). Despite immediate cooling of the core body temperature and aggressive treatment, heat stroke is still the leading cause of death when the climatic temperature is continuously increasing (Kaewput et al., 2021). As global warming intensifies, heat waves are predicted to occur more frequently and are predicted to be longer in duration, while the therapeutic effect of heat stroke is not ideal in the clinic due to the limited knowledge gained on the pathogenesis of heat stroke-induced injury; therefore, the incidence of heat stroke-induced death will increase year by year (Akihiko et al., 2014). The brain is extremely sensitive to temperature alterations, and central nervous system (CNS) abnormalities such as impaired consciousness, delirium, and coma are common in heat stroke patients (Yilmaz et al., 2018). Clinical studies have shown that central nervous system damage occurs in all heat stroke patients, and after discharge from the hospital, 30% of survivors may progress to long-term neurologic damage as sequelae (Argaud et al., 2007; Lawton et al., 2019). Elucidation of the pathophysiological mechanisms of heat stroke-induced brain injury may provide guidelines for heat stroke prevention and therapy.

It is known that heat can pose stress to cell directly, and whether cells can survive or not relies on the cellular homeostasis control mechanisms that, on one hand, eliminate aggregated proteins and damaged organelles, and, on the other hand, synthesize new cellular components and maintain energy supplies. This process is usually described as autophagy and is especially critical to neurons, which are terminally differentiated cells (Liu et al., 2015). Under normal conditions, neuronal autophagy is active and maintained at basal levels, while this level is disrupted and upregulated rapidly under stress, such as nutritional or hormone deficiencies, misfolded protein accumulation, hypoxic stress, and bacterial invasion (Chen et al., 2020). Multiple regulators participate in stress-induced autophagy enhancement in neurons. For example, ischemic anoxia results in a large number of misfolded proteins, and imbalanced Ca^2+^ induced by ischemia and hypoxia promotes neuronal autophagy through inositol-requiring enzyme-1 (IRE1)/tumor necrosis factor receptor associated factor 2 (TRAF2)/JNK and protein kinase R (PKR)-like endoplasmic reticulum kinase (PERK)/eukaryotic initiation factor 2a (eIF2a) signaling pathways (Qi and Chen, 2019). Mitochondrial dysfunction caused by ischemia and hypoxia produces excessive amounts of ROS, which promotes the formation of autophagosomes and transformation of LC3 by activating the expression of Atg4 (Wang et al., 2020). *N*-methyl-D-aspartic acid receptor (NMDA) activation-mediated excitotoxicity enhancement autophagy in striatally damaged neurons resulting from cerebral ischemia may be involved in the JNK signaling pathway (Hou et al., 2019). Heat stress is also a common problem for humans who live or work in extreme environments. Exposure of cultured neuronal cell lines to heat stress results in enhanced autophagy, which further causes brain injury through the activation of apoptosis, as proven by both *in vitro* and *in vivo* experimental research (Zhao et al., 2009; Yi et al., 2017). However, the cellular mechanisms underlying the increased neuronal autophagy in heat stroke are still unknown.

The molecular basis of disordered neuronal autophagy in various brain injury-related diseases has been widely researched. However, most researchers have mainly focused on intraneuronautophagy and have left the investigation of autophagy regulated by cell-cell interactions in a nascent line. Microglia, resident brain macrophages in the central nervous system, survey their environment for damage and are ready to support endangered neurons or interfere with a potential threat to tissue integrity (Hoogland et al., 2015). Danger signals, such as pathogens, damage, and stress, may trigger the transformation of these surveyed microglia to M1 or M2 polarization state. Microglia in M1 polarization state release a variety of proinflammatory factors and neurotoxic mediators to attack neurons, while microglia in M2 polarization state protect neurons by removing cell debris, pruning synapses or supplying nutritive factors such as IGF and BDNF. Accumulated evidence indicates that acute brain injury is often accompanied by the transformation of microglia to M1 polarization state. Shifting the microglial phenotype from M1 to M2 state after the initiation of the healing process or inducing neuroprotective transformation from quiescent microglia could promote nerve repair and regeneration. However, to date, there is no effective strategy that could induce stable M2-type microglial transformation *in vivo*. Therefore, identifying the key molecules in M1-type microglia that damage neurons in neurological diseases and targeting them for intervention is together considered a novel strategy for the development of therapies in diseases related to neuronal injury from the perspective of cell-cell communication.

Exosomes are extracellular vesicles with a diameter of 30–100 nm that have been proven critical to mediate complex and coordinated communication among microglia and neurons. Through exosomes, various genetic materials and proteins are delivered from donor cells to recipient cells. The diverse functions of microglial exosomes rely on the phenotype of microglia, where neuronal damage- or repair-related molecules are packaged in exosomes. For example, neuroprotective microglia-derived exosomes protect the mouse brain from traumatic brain injury by transferring repair-related molecules into neurons to inhibit neuronal inflammation and promote neurite outgrowth (Huang et al., 2018). Exosomes derived from microglia with nerve damage could influence the progression of brain tumors by transferring transcripts of several inflammation-related genes to another dysfunctional microglial cell (Grimaldi et al., 2019). Among the multiple genetic materials in exosomes, microRNAs (miRNAs) are the most investigated and have already been shown to participate in various pathological and physiological processes (Ratajczak and Ratajczak, 2016). Increasing amounts of evidence have demonstrated that neuronal autophagy-related miRNAs can be transferred from microglia to neurons through exosomes and participate in neuronal autophagy regulation in acute brain injury (Li D. et al., 2019). miR-155 is a common autophagy regulator element, and forced expression of miR-155 has been proven to increase autophagic activity in human nasopharyngeal and cervical cancer, as well as cerebral ischemic injury (Wan et al., 2014; Yang et al., 2018). Silencing miR-155 in ischemic cerebral tissues can attenuate cerebral ischemic injury by ameliorating autophagy in brain cells (Wan et al., 2014; Yang et al., 2018). Further studies have indicated that miR-155 could promotes autophagy by targeting several different targets. Among them, Rheb is an indispensable regulator of mTOR activation in response to all stimuli. Rheb is widely expressed in growing or mature neurons and has also been shown to be a autophagy relative target of miR-155 (Wang and Zhang, 2014). mTOR is a well-known negative signal pathway of autophagy, ever research has shown that miR-155 could regulate hypoxia-induced autophagy through mTOR signal pathway (Wan et al., 2014). These studies connect miR-155, Rheb, and mTOR with autophagy regulation. Our preliminary work demonstrated that heat stroke can active microglia to M1 polarization state both *in vivo* and *in vitro*, and during this process, the expression of miR-155 was found to increase dramatically in microglia (Li P. et al., 2019; Li et al., 2021). However, whether miR-155 could be transported from activated microglia to neurons through exosomes and then participate in the regulation of neuronal autophagy through mTOR signal pathway by targeting Rheb in heat stroke is still unknown.

Considering the relationship between miR-155, Rheb, mTOR, and neuronal autophagy, this study attempted to ascertain whether exosome could transfer miR-155 from activated microglia to neuron in the condition of heat stress. Is exosomal miR-155 involved in heat stress-induced neuronal autophagy and how does it influence the neuronal autophagy stimulated by heat stress? In this study, we explore the underlying mechanism of heat stroke-induced brain injury, investigate the crosstalk between microglia and neurons, and evaluate the role of miR-155 in microglial exosome-mediated neuronal autophagy. Our preliminary data showed that microglia could transfer miR-155 to neuron through exosome and then accelerates neuronal autophagy in the condition of heat stress. The underlying mechanisms may include promoting neuronal autophagy by suppressing mTOR pathway *via* targeting Rheb.

## Materials and Methods

### Cell Culture and Treatment

BV-2 microglial cells were purchased from the American Type Culture Collection (ATCC). For the experiments, cells were cultured in Dulbecco’s modified Eagle’s medium (DMEM, Gibco, NY, United States) that included 10% Exo-depleted fetal bovine serum (FBS, SBI), 100 U/ml penicillin, 100 μg/ml streptomycin and 2 mM glutamine (Sigma-Aldrich, St. Louis, MO, United States) at 37°C in a humidified atmosphere of 5% CO_2_. After a 24 h incubation, cells were transfected with miR-155 mimic or its negative control (GenePhama Co., Ltd., Shanghai, China) at a final concentration of 100 nM/ml in Opi-MEM (Life Technologies GmbH, Darmstadt, Germany) using DharmaFECT (GE Healthcare Dharmacon, Lafayette, CO, United States) and then subjected to heat stress for 2 h in a pre-warmed incubator at 42°C, followed by recovery for 6 h at a normal growth temperature of 37°C. The cells and exosomes were collected for further analysis.

N2a cells were purchased from China Infrastructure of Cell Line Resources (Beijing, China) and cultured in DMEM (Gibco, NY, United States) with 10% fetal bovine serum (FBS, HyClone), 100 μg/ml streptomycin and 100 U/ml penicillin (Sigma-Aldrich, St. Louis, MO, United States). Cells were maintained at 37°C in a humidified atmosphere of 5% CO_2_. After a 24 h incubation period, the cells were transfected with or without miR-155 inhibitor or its negative controls (GenePhama Co., Ltd., Shanghai, China) at a final concentration of 200 nM/ml in Opi-MEM (Life Technologies GmbH, Darmstadt, Germany) using DharmaFECT (GE Healthcare Dharmacon, Lafayette, CO, United States), after which the cells were cocultured with or without microglial exosomes for 24 h. Subsequently, the cells were subjected to heat stress for 2 h in a pre-warmed incubator at 42°C, followed by recovery for 6 h at a normal growth temperature of 37°C. The cells were collected for further analysis.

### Transwell Assays

A Transwell system (Corning Co, United States) was used to investigate the communication between neurons and microglial cells. The pore size of the Transwell plate is 0.4 μm, which allows N2a cells and BV-2 cells to share the same medium and substance secreted by cells without direct cell-to-cell contact. BV-2 cells were seeded on Transwell inserts, and N2a cells were cultured in 6-well or 24-well culture plates in the lower compartment according to different assays at a ratio of 3:5. All of them were cultured in DMEM (Gibco, NY, United States) with 10% fetal bovine serum (FBS, HyClone), 100 μg/ml streptomycin, and 100 U/ml penicillin (Sigma-Aldrich, St. Louis, MO, United States) and cultured in a humidified incubator with 5% CO2 at 37°C. After a 24 h incubation, fresh medium with or without GW4869 (20 μM, Sigma-Aldrich, St. Louis, MO, United States) were replaced, after 2 h of GW4869 treatment, cells were subjected to heat stress for 2 h in a pre-warmed incubator at 42°C, followed by a recovery period at the normal growth temperature of 37°C for 6 h. N2a cells were collected for further analysis.

### Microglial Exosome Isolation and Identification

BV-2 microglial cells were cultured in Exo-depleted serum medium for 48 h, and then the supernatant of the culture medium was collected and subjected to sequential ultracentrifugation at 4°C. The supernatant was first centrifuged for 10 min at 300 × *g* to remove free cells and then spun for 20 min at 2000 × *g* to eliminate cell debris. Next, the supernatant was centrifuged at 10000 × *g* for 30 min to remove large vesicles. Afterward, a 0.22 μm filter (Millipore-Sigma, Darmstadt, Germany) was used to further filter particles larger than 200 nm. Finally, ultracentrifugation was performed at 100000 × *g* for 70 min, and exosomes were collected from the pellet and resuspended in PBS for further analysis.

To identify exosomes, we first used transmission electron microscopy (TEM, HT7700; Hitachi, Tokyo, Japan) to observe the morphology of particles in the pellets. Then, Nano Particle Tracking and Zeta Potential Distribution Analyzer (Zetasizer Nanozsp, Malvern, United Kingdom) were used to measure and analyze the size distribution of particles in the pellets. Finally, we used Western blotting to detect specific exosome surface markers, including TSG101, CD63, and CD9.

### Exosome Uptake

To investigate microglia-derived exosome uptake by neurons, a PKH67 kit (green, Sigma-Aldrich, St. Louis, MO, United States) was used according to the manufacturer’s protocol. Briefly, 4 μl of PKH67 solution was added to 1 ml of diluent C, mixed with PKH67 dye and exosome solution, resuspended in 1 ml of diluent C and incubated at room temperature for 5 min. The labeling reaction was terminated with the same volume of Exo-depleted serum. Exosomes were collected by ultracentrifugation at 100000 × *g* for 70 min at 4°C, washed three times with PBS, and resuspended in PBS. These PKH67-labeled exosomes (50 μg/ml) were cocultured with N2a cells for 24 h at 37°C. The cells were washed with PBS and then fixed with 4% paraformaldehyde. The cells were subsequently stained with 4’,6-diamidino-2-phenylindole (DAPI) before observing the uptake of PKH67 exosomes by LSM 780 confocal laser scanning microscopy (400X magnification, Carl Zeiss GmbH, Jena, Germany).

### Real-Time PCR Analysis of miRNAs

Total RNA, including miRNA, was isolated from cultured BV-2 cells, microglial exosomes and N2a cells with TRIzol^®^ reagent (Invitrogen, Carlsbad, CA, United States). The concentration of RNA was measured by a plate reader (Biotek Epoch, Winooski, VT, United States). cDNA was synthesized by MicroRNA First-Strand Synthesis and MiRNA Quantitation Kits (TaKaRa, Tokyo, Japan) according to the manufacturer’s protocol. The expression of miRNA was detected by the Mir-X miRNA qRT-PCR SYBR^§^ Kit (TaKaRa, Tokyo, Japan) with the following cycling conditions: 95°C for 3 min, followed by 40 cycles of 95°C for 15 s, 60°C for 30 s, and 70°C for 10 s. U6 small nuclear RNA served as the internal control for miR-155, and the results were analyzed with the 2^–ΔΔCT^ formula.

### Transmission Electron Microscopy

Cells were harvested by centrifugation, and the pellet was infiltrated with 2.5% glutaraldehyde at 4°C overnight. After washing with PBS three times, the samples were fixed with 1% osmium tetroxide for 1 h and subsequently dehydrated in graded ethanol (30, 50, 70, 90, 96, and 100%). The cells were embedded in Quetol-812 epoxy resin, followed by staining with 2% uranyl acetate and lead citrate at 4°C. Finally, the autophagosome-like vesicles were viewed on a transmission electron microscope (HT7700; Hitachi, Tokyo, Japan).

### Luciferase Reporter Assays

Mut-Rheb-3′UTR and wild-type Rheb-3′UTR dual-luciferase reporter vectors were provided by GenePhama, and N2a cells were cotransfected with miR-155 mimic or its negative control. The pmirGLO-control vector was transfected into N2a cells to monitor transfection efficiency. After 24 h of incubation, we collected the cells and measured the firefly luciferase activity using a dual-luciferase reporter system (Promega, Madison, WI, United States) following the manufacturer’s protocol.

### Lentivirus Transfection

To confirm whether Rheb is involved in the downstream regulation of exosomal miR-155-mediated neuronal autophagy following heat stress, a recombinant lentivirus that overexpresses Rheb (GenePhama Co., Ltd., Shanghai, China) was transfected into N2a cells according to the manufacturer’s protocol. miR-155-upregulated exosome incubation followed by heat stress or rapamycin (100 nM, Sigma, Darmstadt, Germany) treatment was performed. mTOR pathway activation and neuronal autophagy in N2a cells were detected by Western blot and immunofluorescence.

### Immunoblotting

Exosomes or cells were lysed using lysis buffer (Beyotime Biotechnology, China) with freshly added phosphatase inhibitors and protease inhibitors (Roche, Penzberg, Germany). A BCA protein assay kit (ComWin Biotechnology, Beijing, China) was used to detect the protein concentration. Then, 20 μg denatured protein was added to an SDS-PAGE gel and electroblotted onto polyvinylidene difluoride (PVDF) membranes (Bio-Rad, Munich, Germany). Then, 5% fat-free dry milk in Tris-buffered saline with Tween-20 (TBST) buffer was added to block the membranes for 1 h at room temperature. Afterward, the membranes were probed with the following primary antibodies overnight at 4°C: anti-TSG101, anti-CD9, anti-CD63 (Bimake, Houston, TX, United States), anti-LC3 (Abcam, Santa, United States), anti-Beclin1, anti-p-mTOR, anti-mTOR, anti-p-p70s6k, anti-p70s6k, anti-Rheb, and anti-GAPDH (Cell Signaling Technology, Danvers, MA, United States). After washing with TBST, the membranes were incubated with horseradish peroxidase-conjugated secondary antibody for 1 h at room temperature with gentle agitation. Proteins were detected using enhanced chemiluminescence (ECL) reagent (Bio-Rad, Munich, Germany) and scanned by a ChemiDoc MP gel imaging system (Bio-Rad, Munich, Germany). The mean pixel density of each strip was measured by Image Lab (Bio-Rad, Munich, Germany).

### Immunofluorescence Staining

Cells were grown on glass coverslips in 24-pore plates overnight. Then, the cells were washed with PBS and fixed with 4% precooled paraformaldehyde (Beijing Solarbio Science & Technology Co., Ltd., Beijing, China). Cells were permeabilized with 0.5% Triton X-100 (Beijing Solarbio Science & Technology Co., Ltd., Beijing, China) for 15 min. After blocking with goat serum (Zhongshan Golden Bridge Biotechnology, Beijing, China) at room temperature for 60 min, the cells were incubated with MDC (50 μM, Sigma), β-III Tubulin (1:100; mouse, monoclonal, R&D system, United States) or anti-LC3B (1:100; rabbit, monoclonal, Abcam, Santa, United States) antibody overnight at 4°C. The next day, after washing with PBST twice, the cells were incubated with highly cross-adsorbed CF™ 488A-conjugated chicken anti-rabbit IgG secondary antibody (1:250, Sigma-Aldrich, St. Louis, MO, United States) or highly cross-adsorbed CF™ 555-conjugated goat anti-mouse IgG secondary antibody (1:250, Sigma-Aldrich, St. Louis, MO, United States) in the dark for 60 min at 37°C. Nuclei were subsequently counterstained with DAPI (Beyotime Biotechnology, Shanghai, China) for 5 min in the dark. Images were acquired by an LSM 780 confocal laser scanning microscope (400 × magnification, Carl Zeiss GmbH, Jena, Germany) and analyzed by ZEN 2012 light edition software (Carl Zeiss, Jena, Germany).

### Statistical Analysis

Statistical analyses were performed with Prism 5 software (GraphPad, CA, United States). Student’s *t*-test (two groups) or one-way ANOVA (multiple comparisons) was used to assess differences between groups. All the experiments were performed at least three times, and the results are expressed as the means ± standard deviations (SDs). In terms of statistical significance, * means *p* < 0.05, ^**^ means *p* < 0.01, and ^***^ means *p* < 0.001.

## Results

### Microglia Accelerated Heat Stress Induced Neuronal Autophagy

**FIGURE 1:**
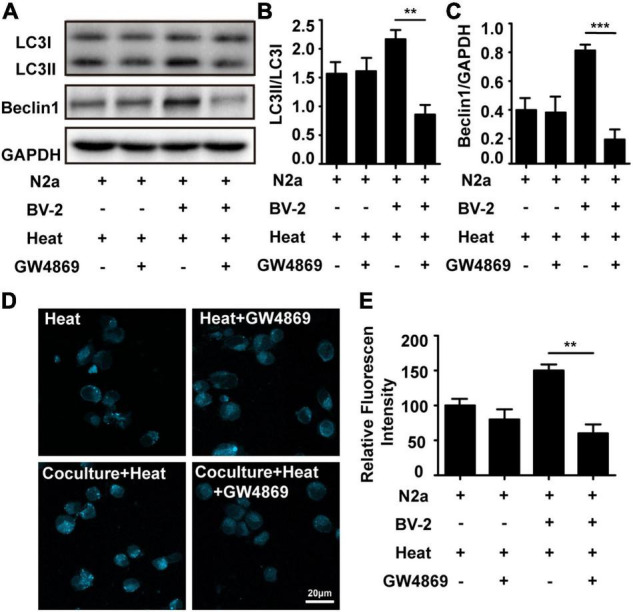
Microglia accelerated heat stress-induced neuronal autophagy. N2a cells cultured alone or cocultured with BV-2 cells were pretreated with or without GW4869 (20 μM, a commonly used inhibitor that can reduce exosomal secretion by decreasing nSMase2 activity) for 1 h and then subjected to heat stress at 42°C for 2 h, followed by a 6 h recovery period at 37°C. **(A–C)** The protein expression of LC3 and Beclin-1 in N2a cells was determined by Western blotting. Densitometric analysis was performed. **(D,E)** The autophagosomes in N2a cells were stained with MDC and subjected to confocal microscopy. Representative images and the analysis are shown. All the data are presented as the means ± SDs of at least three independent experiments. **p < 0.01; ***p < 0.001.

To investigate whether microglia are involved in heat stress-induced neuronal autophagy, a Transwell system was used to coculture microglia and neurons. In this system, N2a cells were seeded in the lower chamber, and BV-2 cells were seeded in the upper chamber. After heat stress treatment, the autophagy-specific proteins LC3 (microtubule-associated protein 1, light chain 3) and Beclin1 (component of phosphatidylinositol-3-kinase complex; an Atg6 homolog) in N2a cells were examined. Usually, LC3 exists in the form of LC3I in the cytoplasm, during autophagy, the conversion of cytosolic LC3I to LC3II is required for autophagic membrane recruitment, and the LC3II levels are related to the extent of autophagosome formation, so the protein expression of LC3II usually used as a measure of autophagy induction. Immunoblotting analysis revealed that neuronal levels of LC3II and Beclin-1 were significantly increased by coculture with BV-2 cells compared with those in the group cultured alone (Figures 1A–C). A typical metabolic feature of autophagosome formation is the formation of acidic vacuolation in the cytoplasm, and monodansylcadaverine (MDC) is a common method used to stain acidic vacuolation, resulting in punctate fluorescence (Cho et al., 2015). Punctuate acidic vacuolation stained with MDCs could be seen in the cytoplasm of heat stress-treated N2a cells cultured alone, and the intensity increased significantly in the coculture group (Figures 1D,E). However, GW4869, a commonly used inhibitor that can reduce exosomal secretion by decreasing nSMase2 activity, partly reversed heat stress-induced autophagy in N2a cells in the coculture system but did not influence autophagy in N2a cells cultured alone (Figures 1A–E). All the results of negative control present in Supplementary Figure 2. Besides autophagy, we also evaluated the viability of N2a cells through CCK-8 assay. Result showed that N2a cell viability was decreased by heat stress and further down by coculturing with BV2, while GW4869 pretreatment could significantly improve N2a cells viability which decreased by coculturing with BV2 cell under heat stress condition (Supplementary Figure 1A). Taken together, these results indicated that microglia contributed to heat stress-induced neuronal autophagy and that during this process, exosomes may play a critical role.

### Microglial Exosome Identification and Absorption

**FIGURE 2:**
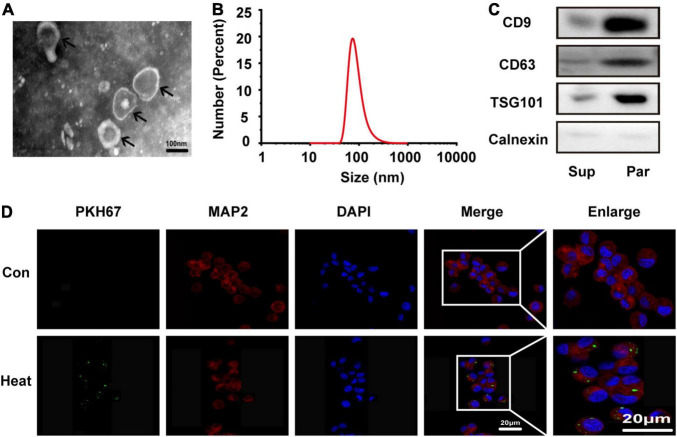
Microglia-derived exosomes were characterized and taken up by N2a cells. Exosomes were extracted from microglial culture supernatant by ultracentrifugation. **(A)** Morphology was observed by transmission electron microscopy (TEM). **(B)** The size distribution was measured by a Nanozsp tracking analyzer. **(C)** Biomarkers of exosomes, including CD9, CD63, TSG101, and Calnexin, were analyzed by Western blotting. **(D)** N2a cells were co-incubated with or without PKH67-labeled microglial exosomes (50 μg/ml) for 24 h, the cells were then stained with anti-MAP2 and DAPI, and microglial exosome uptake was detected *via* confocal microscopy. Representative and enlarged images are shown. Sup refers to microglial supernatant, Par refers to microglia-generated particles.

To further investigate whether microglia accelerate heat stress-stimulated neuronal autophagy through exosomes, we extracted exosomes from microglial culture supernatant by ultracentrifugation. The morphology, size distribution and characteristic markers of exosomes were identified by transmission electron microscopy, nanoparticle tracking analysis and Western blotting. As shown in Figures 2A,B, a bilayer membrane vesicle with round-shaped morphology and a diameter range of 30–120 nm that typically peaked at 81 nm was detected. Differently expressed biomarkers, including CD9, CD63, TSG101, and Calnexin, further confirmed that exosomes were successfully purified from microglial cultures (Figure 2C). To further verify whether BV2 exosomes could be taken up by N2a cells, PKH67-labeled BV2 exosomes were added to the N2a cell culture medium. After 24 h, fluorescence microscopy was used to examine exosome uptake by N2a cells immunopositive for MAP2 in the cytoplasm. N2a cells without exosomes treatment as the control group. The results showed that green fluorescence-labeled exosomes colocalized with red fluorescence-labeled N2a cells (Figure 2D), confirming that BV2 exosomes could be engulfed by N2a cells.

### Exosomes Derived From Heat Stress-Treated Microglia (Heat-EXOs) Greatly Enhanced Neuronal Autophagy

**FIGURE 3:**
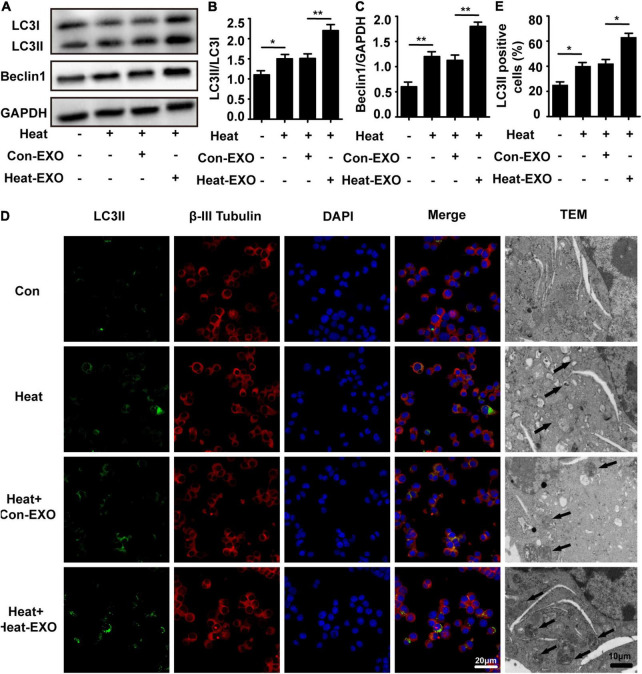
Exosomes derived from heat stress-treated microglia greatly enhanced neuronal autophagy. N2a cells cultured alone or incubated with exosomes (50 μg/ml) derived from sham or heat-stressed microglia for 24 h to allow enough exosomal uptake and then subjected to heat stress at 42°C for 2 h, followed by a 6 h recovery period at 37°C. **(A–C)** The protein expression of LC3 and Beclin-1 in N2a cells was determined by Western blotting. Densitometric analysis was performed. **(D,E)** The location of β-III Tubulin, LC3II, and autophagosomes in the cytoplasm of N2a cells were examined *via* confocal microscopy and TEM. Representative images and the analysis are shown. Con-EXO refers to exosome extracted from microglia which receives no treatment, Heat-EXO refers to exosome extracted from microglia which receives heat stress treatment. All the data are presented as the means ± SDs of at least three independent experiments. *p < 0.05; **p < 0.01.

To further confirm that microglial exosomes are involved in heat stress-stimulated N2a cell autophagy. We extracted exosomes from BV-2 cells that were subjected to heat stress or sham treatment, and the autophagy of N2a cells was assessed by Western blot, immunofluorescence staining of LC3II and transmission electron microscopy after exosome and heat stress treatment. As shown in Figures 3A–E, compared to that of sham-treated microglial exosomes, the administration of exosomes that were subjected to heat stress treatment greatly increased the number of N2a cells with large accumulations of LC3II. A similar pattern was observed with transmission electron microscopy, which detected autophagosomes in the cytoplasm of N2a cells following heat stress treatment. Besides autophagy, we also evaluated the viability of N2a cells through CCK-8 assay. Result showed that N2a cell viability was decreased by heat stress and further down by coculturing with exosomes derived from heat stress-activated microglia (Supplementary Figure 1B). Taken together, these findings provide evidence that exosomes extracted from heat stress-treated microglia accelerate heat-induced neuronal autophagy.

### miR-155 in N2a Cells Increased After Incubation With BV2 Cell Exosomes

**FIGURE 4:**
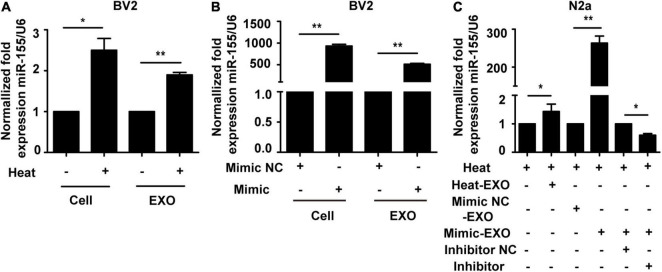
Incubation with microglial exosomes increases miR-155 expression in N2a cells. **(A)** BV-2 cells were incubated at 37°C (control) or were subjected to heat stress at 42°C for 2 h, followed by a recovery period at 37°C for 6 h. The expression of miR-155 in BV-2 cells and exosomes was measured by qRT-PCR and normalized to U6 expression. **(B)** BV-2 cells were transfected with miR-155 mimic or miR-155 mimic negative control for 24 h, and then miR-155 expression in BV-2 cells and their exosomes was measured by qRT-PCR and normalized to U6 expression. The results are presented as the fold change with respect to the negative control. **(C)** N2a cells were pretreated with or without miR-155 inhibitor or its negative control and then incubated with exosomes (50 μg/ml) derived from BV-2 cells that were subjected to heat stress, miR-155 mimic, or miR-155 mimic negative control transfection. Afterward, the cells were subjected to heat stress at 42°C for 2 h, followed by a 6 h recovery period at 37°C. Then, the expression of miR-155 in N2a cells was examined by qRT-PCR and normalized to U6 expression. NC refers to negative control of the treatment factors. All the data are presented as the means ± SDs of at least three independent experiments. *p < 0.05; **p < 0.01.

Exosomes are well-known to be enriched with miRNAs and able to deliver miRNA from host cells to target cells to further regulate the function of recipient cells (Song et al., 2019). To explore the underlying mechanism of microglial exosome-mediated neuronal autophagy in the context of heat stress, we examined the expression of miR-155 in microglia, microglial exosomes and neurons. Total RNA of microglia or their exosomes was collected, and qRT-PCR was conducted following heat stress treatment. Our results indicated that heat stress could promote the expression of miR-155 in both microglia and microglial exosomes (Figure 4A). To further investigate the function of miR-155, we overexpressed miR-155 in microglial exosomes by transfecting miR-155 mimic into microglia, and then the transfection efficacy was determined by qRT-PCR. The relevant results showed that compared to that in the cells transfected with negative controls, the expression of miR-155 both in microglia and microglial exosomes significantly increased, indicating effective transfection (Figure 4B). Then, we incubated exosomes derived from microglia that were subjected to heat stress treatment or miR-155 overexpression with N2a cells pretreated with miR-155 inhibitor or its negative control. Afterward, N2a cells were subjected to heat stress at 42°C for 2 h, followed by a 6 h recovery period at 37°C, miR-155 expression in N2a cells was evaluated by qRT-PCR. The results showed that exosomes derived from microglia that were subjected to heat stress treatment or miR-155 overexpression significantly increased miR-155 expression in N2a cells, while pretreatment with miR-155 inhibitor significantly decreased miR-155 expression, which was upregulated by miR-155-upregulated exosomes (Figure 4C) in N2a cells. Taken together, these results indicated that miR-155 could be transported from microglia to neurons through exosomes.

### miR-155 Is Critical in Microglial Exosome-Mediated Neuronal Autophagy Following Heat Stress

**FIGURE 5:**
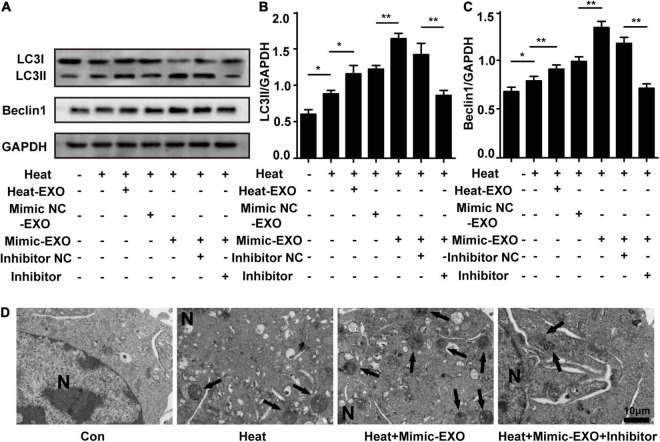
miR-155 plays a critical role in the regulation of heat stress-induced neuronal autophagy by microglial exosomes. N2a cells were pretreated with or without miR-155 inhibitor or its negative control and then incubated with exosomes (50 μg/ml) derived from BV-2 cells that were subjected to heat stress, miR-155 mimic, or miR-155 mimic negative control transfection. Finally, the cells were subjected to heat stress at 42°C for 2 h, followed by a 6 h recovery period at 37°C. **(A–C)** The protein expression of LC3 and Beclin-1 in N2a cells was determined by Western blotting. Densitometric analysis was performed. **(D)** Autophagosomes in the cytoplasm of N2a cells were observed by TEM. Representative images are shown. All the data are presented as the means ± SDs of at least three independent experiments. *p < 0.05; **p < 0.01.

To further investigate the function of miR-155 in microglial exosomes mediating neuronal autophagy following heat stress, N2a cells were cocultured with exosomes that were harvested from microglia subjected to heat stress or transfected with miR-155 mimic, and the expression levels of neuronal autophagy were examined after cells were subjected to heat stress treatment. Immunoblotting analysis revealed that compared to the heat stress-only group, the expression levels of LC3II and Beclin1 in neurons were increased after incubation with heat stress-treated microglial exosomes and were further upregulated by coculturing with miR-155-upregulated exosomes, while miR-155 inhibitor pretreatment abolished the increase in LC3II and Beclin1, which was detected by incubating miR-155-upregulated exosomes with N2a cells (Figures 5A–C). Autophagosomes detected by transmission electron microscopy obtained similar results to those detected by Western blotting. As shown in Figure 5D, compared with the heat stress-only group, the group treated with miR-155-upregulated exosomes demonstrated a greater number of autophagosomes. However, pretreatment with the miR-155 inhibitor significantly decreased the number of autophagosomes in N2a cells (Figure 5D). These results were also verified by immunolocalization of LC3II in N2a cells with confocal microscopy (Supplementary Figure 3). Besides autophagy, we also evaluated the viability of N2a cells through CCK-8 assay. Result showed that N2a cell viability was decreased by heat stress and further down by coculturing with exosomes derived from microglia which overexpressed with miR-155, while miR-155 inhibitor pretreatment could significantly improve N2a cells viability which decreased by overexpression of miR-155 under heat stress condition (Supplementary Figure 1C). According to the above results, as the content of miR-155 increased, the autophagy of N2a cells increased, and this effect could be partly reversed by the miR-155 inhibitor. This phenomenon indicated that miR-155 is critical in microglial exosome-mediated neuronal autophagy following heat stress.

### Exosomal miR-155 Accelerated Heat Stress-Induced Neuronal Autophagy by Directly Targeting Rheb

**FIGURE 6:**
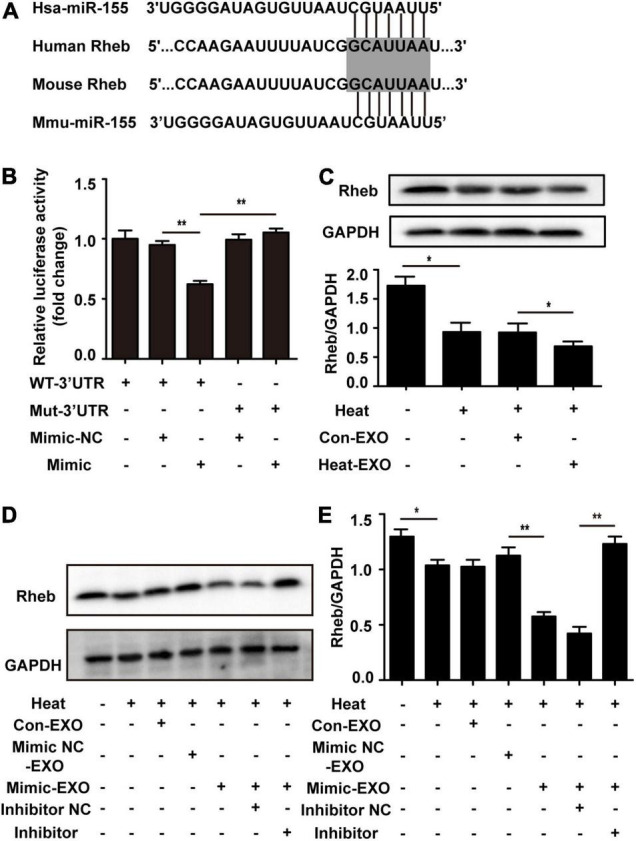
Microglial exosomal miR-155 enhanced neuronal autophagy by targeting Rheb. **(A)** A potential binding site for human and mouse miR-155 in the Rheb 3′UTR. The seed region is highlighted. **(B)** The luciferase reporter plasmids carrying the WT or Mut 3′UTR of Rheb were cotransfected with either the miR-155 mimic or miR-155 mimic-NC into N2a cells, after 24 h, luciferase activity was detected. **(C)** N2a cells which cultured alone or incubated with exosomes (50 μg/ml) derived from sham or heat exposure treated microglia for 24 h to allow enough exosomal taken-up, and then subjected to heat exposure at 42°C for 2 h, followed by a 6 h recovery period at 37°C. The protein expression of Rheb in N2a cells was determined by Western blotting. Densitometric analysis was performed. **(D)** N2a cells pretreated with or without miR-155 inhibitor or its negative control, and then incubated with exosomes (50 μg/ml) derived from BV-2 cells which do not received any treatment, miR-155 mimic or miR-155 mimic negative control transfection, respectively. After that, cells were subjected to heat exposure at 42°C for 2 h, followed by 6 h recovery period at 37°C. Then the protein expression of Rheb in N2a cells was determined by Western blotting. **(E)** Densitometric analysis was performed. All data are presented as mean ± SD of three independent experiments at least. *p < 0.05; **p < 0.01.

In order to investigate the mechanism of the exosomal miR-155-mediated neuronal autophagy, we scanned the potential target genes of miR-155 on TargetScan or miRanda and then determined the expression of several miR-155 targets that are related to brain. The expression of TBR1, Rheb, and BDNF was detected in cultured neurons treated with Heat-EXO. The results indicated that treatment with Heat-EXO significantly reduced the expression of Rheb (Supplementary Figure 2). To explore the mechanism of miR-155-mediated neuronal autophagy, we predicted the potential target genes of miR-155 on TargetScan and miRanda. The 3′UTR of Rheb mRNA in mice has putative binding sites for miR-155, and the binding site seems to be highly conserved in humans (Figure 6A). As such, luciferase reporter assays were performed to verify whether Rheb is a direct target of miR-155. As shown in Figure 6B and Supplementary Figure 5A, the miR-155 mimic strongly repressed luciferase activity in cells transfected with the Rheb 3′-UTR, while cells transfected with the Rheb 3′-UTR mutant seemed to have no difference in luciferase activity compared to the control group. Our results suggested that Rheb is the direct target of miR-155. To further confirm the miR-155 function in our research by targeting Rheb, N2a cells were cocultured with exosomes derived from microglia subjected to sham or transfected with miR-155 mimic. After being subjected to heat stress treatment, the cells were harvested for Western blot analysis. Our results indicated that as the expression of miR-155 increased in the exosomes, Rheb expression in N2a cells decreased. Pretreatment with the miR-155 inhibitor in N2a cells significantly rescued Rheb expression, which was repressed by miR-155-upregulated exosomes (Figures 6C–E and Supplementary Figure 5B). These results revealed that Rheb is a functional target of miR-155 in microglial exosome-mediated neuronal autophagy following heat stress.

### Overexpression of Rheb Ameliorates Microglial Exosomal miR-155-Evoked Neuronal Autophagy by Promoting the Activity of mTOR Signaling

**FIGURE 7:**
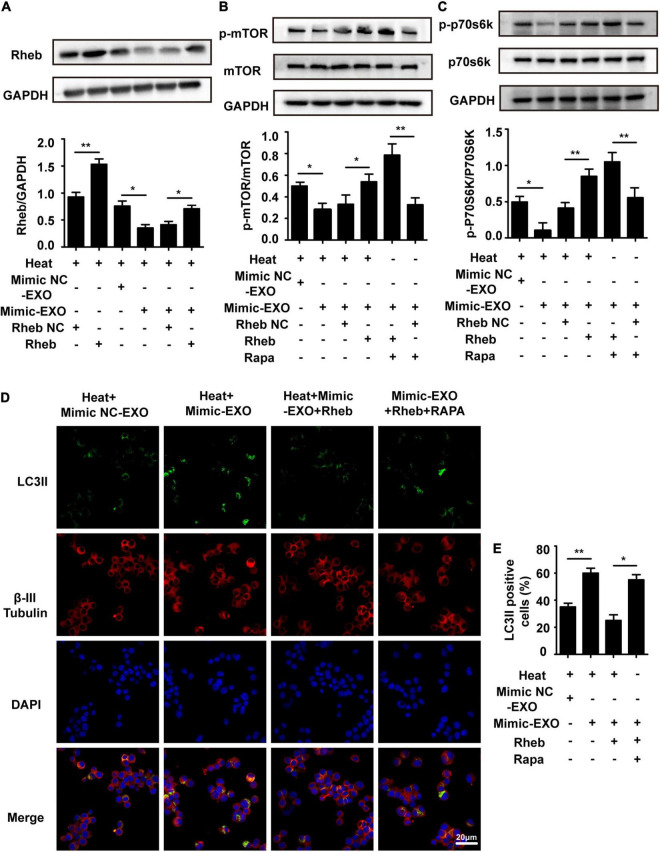
Overexpression of Rheb ameliorates microglial exosomal miR-155-induced neuronal autophagy by promoting mTOR signaling. N2a cells were transfected with or without a recombinant lentivirus that overexpressed Rheb and then incubated with miR-155-upregulated microglial exosomes (50 μg/ml) alone or together with 100 nM Rapa. Afterward, the cells were subjected to sham or heat stress at 42°C for 2 h, followed by a 6 h recovery period at 37°C. **(A–C)** The expression of Rheb and activation of mTOR and p70s6k with antibodies against Rheb, phospho-mTOR, phospho-p70s6k, mTOR, and p70s6k was determined by Western blotting. Densitometric analysis was performed. **(D,E)** The immunolocalization of LC3II and β-III Tubulin in the cytoplasm of N2a cells was examined *via* confocal microscopy. Representative images and the analysis are shown. Rapa is rapamycin, it could activate autophagy by specifically suppressing mTOR signal pathway in cells. All the data are presented as the means ± SDs of at least three independent experiments. *p < 0.05; **p < 0.01.

To further confirm the involvement of Rheb in microglial exosome-mediated neuronal autophagy regulation, a recombinant lentivirus that overexpressed Rheb in N2a cells (Rheb+) was used to evaluate the effect of overexpressing Rheb on neuronal autophagy in heat stress-stimulated N2a cells with miR-155-upregulated exosome preconditioning. The effectiveness of Rheb overexpression was confirmed by Western blotting. Incubation with miR-155-upregulated microglial exosomes significantly decreased Rheb expression in heat-stressed N2a cells compared with the negative control cells, while overexpression of Rheb abolished the suppressive effect of miR-155 on Rheb (Figure 7A). Immunofluorescence analysis revealed that LC3II accumulation increased with miR-155-upregulated exosomes and heat stress treatment but decreased with transfection of recombinant lentivirus overexpressing Rheb in N2a cells (Figure 7D). All of the above results confirm that Rheb is involved in microglial exosome-mediated neuronal autophagy regulation. Mammalian target of rapamycin (mTOR) is a key regulator of cell survival, including growth, autophagy, and translation (Laplante and Sabatini, 2012). Accumulating amounts of evidence have proven that Rheb affects autophagy mainly by regulating the mTOR signaling pathway (Moon et al., 2019). Therefore, we checked the roles of mTOR signaling in Rheb-mediated exosomal miR-155-regulated neuronal autophagy following heat stress. We found that the expression levels of p-mTOR and p-P70S6K were significantly suppressed by miR-155-upregulated exosomes and heat stress treatment, while overexpression of Rheb blocked the suppressive effect of miR-155 on mTOR signaling, reflected by increased expression of p-mTOR and p-P70S6K in the Rheb overexpression group (Figures 7B,C). To further confirm Rheb-mTOR pathway in exosomal miR-155-regulated neuronal autophagy, the mTOR inhibitor rapamycin was used to specifically suppress mTOR signaling in N2a cells. Neuronal autophagy was evaluated by immunolocalization of LC3II in N2a cells pretreated with miR-155-upregulated exosomes and Rheb overexpression treatment. As shown in Figures 7D,E, the addition of rapamycin significantly reversed the LC3II level, which decreased in response to Rheb overexpression in N2a cells, indicating that mTOR is involved in miR-155-Rheb-regulated neuronal autophagy following heat stress. In addition, the result of control group in which rapamycin-treated N2a cells without any other treatment has been presented in Supplementary Figure 6. Besides, we also evaluated the viability of N2a cells through CCK-8 assay. Result showed that N2a cell viability was significantly decreased by exosomes overexpressed with miR-155, while upregulated Rheb could significantly improve N2a cells viability which decreased by coculturing with exosome overexpressed with miR-155 under heat stress condition, pretreatment with Rapa also could save the viability of N2a cells which reduced by coculturing with miR-155 upregulated exosomes (Supplementary Figure 1D). Taken together, these results revealed that microglial exosomal miR-155 accelerated heat stress-induced neuronal autophagy mainly through suppressing the Rheb-mTOR signaling pathway.

## Discussion

**FIGURE 8:**
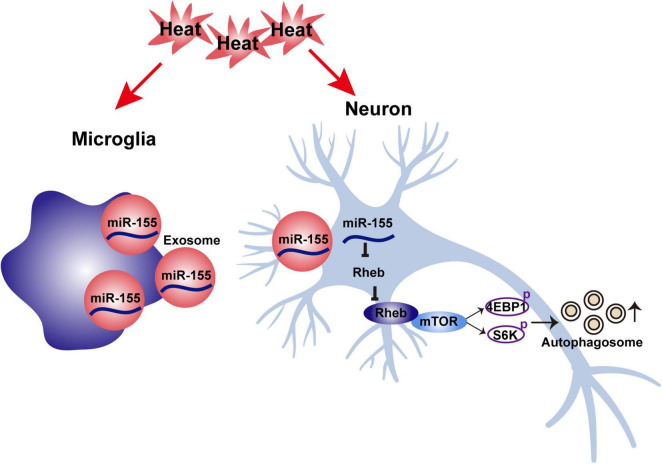
Schematic illustration of the mechanism underlying the effects of microglia-derived exosomal miR-155 on neuronal autophagy after heat stress.

Neuronal autophagy dysfunction is one of the main reasons for heat stroke-induced brain injury, and activated microglia play a critical role in modulating neuronal autophagy either in direct or indirect ways. In the present study (as shown in Figure 8), we found that activated microglia accelerate heat stress-induced neuronal autophagy through transmission of exosomes to neurons. During this process, increased expression of miR-155 was detected in microglia, microglial exosomes and neurons cocultured with microglial exosomes. Moreover, overexpression of miR-155 in microglia further enhanced heat stress-induced neuronal autophagy *via* their transfer by exosomes. However, inhibition of miR-155 in neurons could partly abolish the increased neuronal autophagy caused by coculture with microglial exosomes edited with miR-155 overexpression, indicating that miR-155 is a key molecule that participates in heat stress-induced neuronal autophagy through microglial exosomes. Further research demonstrated that these effects of miR-155 were exerted by targeting Rheb, thus inhibiting the activity of mTOR signaling. Therefore, our results suggest that miR-155 may be a promising target for intervention in neuronal autophagy after heat stroke.

GW4869 is the most widely used pharmacological agent for blocking exosome generation, it could inhibit the ceramide-mediated inward budding of multivesicular bodies (MVBs) and release of mature exosomes from MVBs. Our research prove GW4869 could reversed heat stress-induced autophagy in N2a cells in the coculture system but did not influence autophagy in N2a cells cultured along, indicated microglial exosome participates in heat stress-induced neuronal autophagy. In this result, we also observe an interesting phenomenon in which the autophagy level in the Coculture + Heat + GW4869 group seems much lower than both the Heat group and Coculture + Heat group. Ever researches have reported that proinflammatory cytokines secreted by M1 microglia could cause neuronal autophagy, while anti-inflammatory drugs could alleviate disease progression by regulating neuronal autophagy (Du et al., 2017; Yerra et al., 2017). Recently, a number of studies have begun to examine the effects of GW4869 on immune cell function and as potential anti-inflammatory agents. Essandoh et al. (2015) found that block of exosome generation with GW4869 dampens the sepsis-induced inflammation and cardiac dysfunction, in their study, they discovered pretreatment with GW4869 significantly impaired release of pro-inflammatory cytokines such as TNF-a, IL-1β, and IL-6 which stimulated by LPS in RAW264,7 macrophages. Kumar et al. (2019) demonstrated that use GW4869 inhibit neutral sphingomyelinase could alleviate LPS-induced microglia activation and neuroinflammation after experimental Traumatic Brain Injury (TBI), in their study, they found LPS could induce BV2 microglia cells and primary microglia to secrete pro-inflammatory mediators, while GW4869 pretreatment could significantly downregulate expression of TNF-a, IL-1β, IL-6, iNOS, and CCL2 which stimulated by LPS in microglia. Furthermore, a recent study proved that inhibition of exosome release by GW4869 could alter the activation of microglia and pro-inflammatory cytokines secretion in TBI mice (Hu et al., 2022). Combine the effect of neuroinflammation on neuronal autophagy and the anti-inflammation effect of GW4869 on microglia caused neuroinflammation in acute brain injury model, we speculated that the reason of neuronal autophagy in Coculture + Heat + GW4869 group seems much lower than Coculture + Heat group is GW4869 decreased the level of pro-inflammatory cytokines which no matter transmitted by exosome or paracrine action from microglia to neuron.

In recent years, as potent vehicles of cell-cell communication, exosomes have received increasing amounts of attention in the fields of immunology, tumor biology, neurobiology, and others. Moreover, increasing numbers of studies have indicated that nearly all types of cells secrete exosomes, and these vesicles can be isolated from conditional cell culture medium and various extracellular bodily fluids, such as blood, sweat, urine, and cerebrospinal fluids, by ultracentrifugation or commercial kits (Wu et al., 2017). Characteristic biomarkers expressed on the membrane of exosomes can reflect their cellular source origin and destination for delivery, and the lipid bilayer structure protects enveloped molecules well and allows exosomes to easily cross the blood-brain barrier (Pfrieger and Vitale, 2018). Through receptor-mediated adhesion to the target cellular plasma membrane, exosomes can be internalized by target cells through cell type-specific phagocytosis, endocytosis or direct membrane fusion, thereby finishing message transmission between donor and recipient cells (Hou et al., 2020). Given the above critical roles, exosome-mediated research on pathology and therapeutic strategies involves various diseases; among them, microglia with nerve damage-derived exosomes play significant roles in the development and progression of CNS diseases (Paolicelli et al., 2019). For example, in neurodegenerative disease, exosomes derived from Aβ- or alpha-synuclein-activated microglia deliver misfolded proteins and membrane tumor necrosis factor alpha (TNF-alpha) to neurons, which cause neuronal dysfunction and death (DeLeo and Ikezu, 2018; Xia et al., 2019). In addition, lipopolysaccharide (LPS)-activated microglial exosomes express high levels of TNF-inducible protein 3, and knockdown of TNFAIP3 promotes neurological output by reversing neuronal apoptosis (Zhang et al., 2021). In the present study, we observed that exosomes derived from microglia with nerve damage promote heat stress-induced neuronal autophagy, thereby participating in the regulation of heat stroke-induced brain injury. Together, microglial exosomes play a significant role in the progression of CNS diseases, including heat stroke.

Among the multiple functional cargos transported by exosomes, miRNAs are the most investigated and have been shown to compose a class of important molecules with definite functions in regulating gene expression post-transcriptionally. Transmission through exosomes is considered to be an important mechanism for the stable existence of miRNAs in the circulation (Cheng et al., 2014). Increasing amounts of evidence indicate that exosomes can deliver mature miRNAs to recipient cells and then play critical roles in many physiological and pathological processes through degradation of target mRNAs in recipient cells. We found that miR-155 levels increased in heat-stressed microglia, microglial exosomes, and N2a cells (recipient cells). miR-155 is a typical autophagy inducer highly expressed in lung, heart and cerebrum tissues (Faraoni et al., 2009; Wan et al., 2014; Zhang et al., 2017). Increased expression of miR-155 promotes neuronal autophagy in the diseased brain, which is beneficial to many neurodegenerative diseases, such as Alzheimer’s disease and Parkinson’s disease, by eliminating deposited toxic proteins, such as APP/Aβ and α-synuclein. However, in acute brain injury models, such as ischemia-reperfusion or traumatic brain injury, overexpression of miR-155 in neurons is usually accompanied by the aggravation of brain injury. However, inhibition of miR-155 in injury models seems to protect the brain by decreasing neuronal autophagy (Chen et al., 2018; Yang et al., 2018). Our results confirm a similar viewpoint in heat stroke from the perspective of exosomes. In the present research, we demonstrated that miR-155 evoked by acute heat stress in microglia could accelerate autophagy in neurons *via* their transfer by microglial exosomes and that knocking down miR-155 within neurons alleviates heat stress-induced neuronal autophagy. These results indicate that the increased miR-155 in microglial exosomes may play a significant role in the injured brain after heat stroke.

Studies have shown that miR-155 participates in regulating hypoxia-induced autophagy by targeting the key protein of the mTOR pathway, a well-known negative regulator of autophagy (Wan et al., 2014). Ras homolog enriched in brain (Rheb), a small guanosine triphosphate (GTP) enzyme that belongs to the Ras superfamily, is an indispensable regulator of mTOR activation in response to all stimuli (Wang and Zhang, 2014). In the present study, after coculturing miR-155-edited microglial exosomes with N2a cells, we observed a significant inverse correlation between miR-155 and Rheb expression in heat-stressed N2a cells. Both bioinformatics analysis and luciferase reporter assays further verified that Rheb is a target of miR-155. Substantial amounts of data demonstrated that Rheb can bind to GTP to form the GTP-Rheb complex, thereby activating mTORC1 to promote substrate recruitment. Then, two specific downstream effectors of mTOR, eukaryotic initiation factor 4E-binding protein (4E-BP1) and p70 ribosomal S6 protein kinases (S6Ks), were phosphorylated, and autophagy was negatively regulated (Hay and Sonenberg, 2004). Rheb-mTOR is the key signaling pathway component that regulates cognitive impairment caused by autophagy dysfunction in the hippocampus during brain aging. Upregulating Rheb could activate the mTOR signaling pathway, thereby decreasing hippocampal neuronal autophagy and improving the learning and memory capacity of aging rats (Wang et al., 2018). Inhibition of mTORC1 with rapamycin reversed the increased neuronal activity in the spine, while overexpression of Rheb induced obvious phosphorylation of mTOR, S6, and 4EBP1 in a chronic constriction injury mouse model (Ma et al., 2020). In the present study, the activity of mTOR in heat-stressed neurons was suppressed by microglial exosomal miR-155 by targeting Rheb. Overexpression of Rheb by a recombinant lentivirus blocks the suppressive effect of microglial exosomal miR-155 on the mTOR signaling pathway. Our study confirmed that Rheb plays a critical role in heat stress-induced neuronal autophagy.

Although we preliminarily revealed the mechanism of microglia-neuron communication in the brain after heat stroke, two limitations of this study should be considered. One limitation is lack of animal experiments to support the specific mechanisms underlying neuronal autophagy increases by microglial exosomal miR-155. Therefore, it is not clear that whether exosomal miR-155 could be transferred from microglia to neuron *in vivo* and whether exosomal miR-155 could regulate mTOR signal pathway by targeting Rheb in heat stroke animal model. We will further confirm our conclusions by performing heat stroke animal model experiments in our future studies. The other limitation is that the cells we used in this research were not primary cells. Exosome extraction requires a large amount of cell culture medium, which is difficult to achieve by culturing primary cells.

## Conclusion

In this study, we investigated the effect and mechanism of microglial exosomal miRNAs in regulating brain injury after heat stroke by controlling neuronal autophagy. We found that activated microglia accelerate heat stress-induced neuronal autophagy through transmission of exosomal miR-155 to neurons, with the subsequent Rheb-mTOR signaling pathway suppressed and neuronal autophagy enhanced. Regardless of the detailed mechanism, the data presented in this research indicated that miR-155 may be a promising target for intervention in neuronal autophagy after heat stroke.

## Data Availability Statement

The raw data supporting the conclusions of this article will be made available by the authors, without undue reservation.

## Author Contributions

X-SY and PL designed the research. PL, XL, ZL, G-LH, T-TS, X-TY, Z-ZW, Y-LT, and X-QL performed the experiments. PL, XL, and ZL analyzed the data. PL and X-SY wrote the manuscript. All authors have reviewed the manuscript and approved the submitted version.

## Conflict of Interest

The authors declare that the research was conducted in the absence of any commercial or financial relationships that could be construed as a potential conflict of interest.

## Publisher’s Note

All claims expressed in this article are solely those of the authors and do not necessarily represent those of their affiliated organizations, or those of the publisher, the editors and the reviewers. Any product that may be evaluated in this article, or claim that may be made by its manufacturer, is not guaranteed or endorsed by the publisher.

## Abbreviations

Aβ: β-amyloid
APP: β-amyloid precursor protein
Atg4: autophagy related 4
BDNF: brain derived neurotrophic factor
CD9: cluster of differentiation 9
CD63: cluster of differentiation 63
CNS: central nervous system
eIF2a: eukaryotic initiation factor 2a
EXO: exosome
4E-BP1: 4E-binding protein
GTP: guanosine triphosphate
HS: heat stress
IGF: insulin-like growth factor
IRE1: inositol-requiring enzyme-1
JNK: Jun-N-terminal kinase
LC3: light chain 3
LPS: lipopolysaccharide
MAP2: microtubule-associated protein 2
MDC: monodansylcadaverine
mTOR: mammalian target of rapamycin
mTORC1: mTOR complex
NMDA: *N*-methy 1-D-aspartic acid receptor
P70S6Ks: p70 ribosomal S6 protein kinases
PERK: protein kinase R-like endoplasmic reticulum kinase
PKR: protein kinase R
ROS: reactive oxygen species
Rheb: Ras homolog enriched in brain
TNF-alpha: tumor necrosis factor alpha
TNFAIP3: TNF-inducible protein 3
TRAF2: tumor necrosis factor receptor associated factor 2
TSG101: tumor susceptibility gene 101 protein.
