# Supplementary material for: Increased miR-155 in Microglial Exosomes Following Heat Stress Accelerates Neuronal Autophagy via Their Transfer Into Neurons
Source: Front Cell Neurosci. 2022 May 11;16:865568. doi: 10.3389/fncel.2022.865568 (PMC9132214; doi:10.3389/fncel.2022.865568)
Supplement: Supplementary file 1 [file Data_Sheet_1.pdf]

Supplementary Material

Supplementary Figures

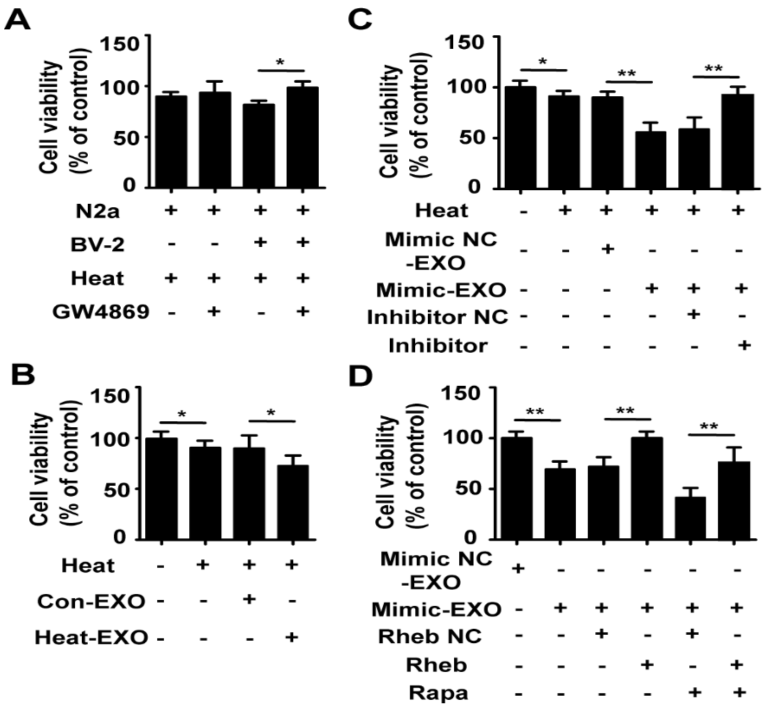

Figure S1: Cell viability attached to Figure1 (A), Figure3(B), Figure5(C) and Figure7(D). (A) N2a cells cultured alone or cocultured with BV-2 cells were pretreated with or without GW4869 (20 µM) for 1 h and then subjected to heat stress at 42°C for 2 h, followed by a 6 h recovery period at 37°C. Viability of N2a cells was determined by CCK8. **(B)** N2a cells cultured alone or incubated with exosomes derived from sham or heat-stressed microglia for 24 h to allow enough exosomal uptake and then subjected to heat stress at 42°C for 2 h, followed by a 6 h recovery period at 37°C. Cell viability was determined by CCK8. **(C)** N2a cells were pretreated with or without miR-155 inhibitor or its negative control and then incubated with exosomes derived from BV-2 cells that were subjected to miR-155 mimic or miR-155 mimic negative control transfection. Finally, the cells were subjected to heat stress at 42°C for 2 h, followed by a 6 h recovery period at 37°C. Cell viability was determined by CCK8. **(D)** N2a cells were transfected with or without a recombinant lentivirus that overexpressed Rheb and then incubated with miR-155-upregulated microglial exosomes alone or together with 100 nM Rapa. Afterward, the cells were subjected to heat stress at 42°C for 2 h, followed by a 6 h recovery period at 37°C. Cell viability was determined by CCK8. All the data are presented as the means±SDs of at least three independent experiments. *p<0.05; **p<0.01.

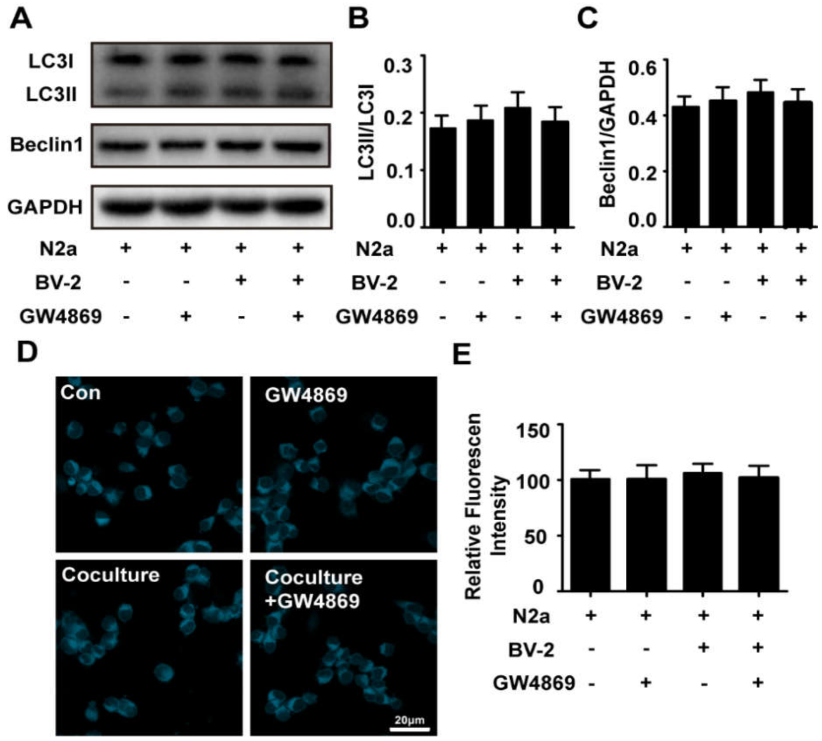

Figure S2: Negative control of Figure1. N2a cells cultured alone or cocultured with BV-2 cells and then treated with or without GW4869 (20 μM, a commonly used inhibitor that can reduce exosomal secretion by decreasing nSMase2 activity) for 1 h. **(A-C)** The protein expression of LC3 and Beclin-1 in N2a cells was determined by Western blotting. Densitometric analysis was performed. **(D-E)** The autophagosomes in N2a cells were stained with MDC and subjected to confocal microscopy. Representative images and the analysis are shown. are shown. All the data are presented as the means±SDs of at least three independent experiments.

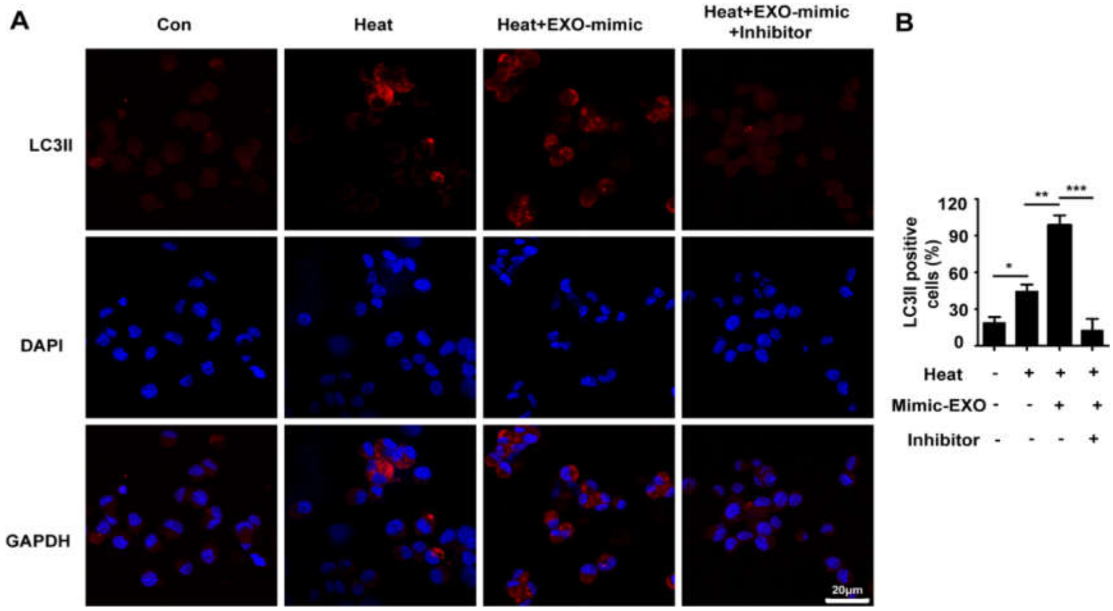

Figure S3: miR-155 plays a critical role in the regulation of heat stress-induced neuronal autophagy by microglial exosomes N2a cells were pretreated with or without miR-155 inhibitor or its negative control and then incubated with exosomes (50μg/ml) derived from BV-2 cells that were subjected to heat stress, miR-155 mimic or miR-155 mimic negative control transfection. Finally, the cells were subjected to heat stress at 42°C for 2 h, followed by a 6 h recovery period at 37°C. **(A-B)** The location of LC3II in the cytoplasm of N2a cells were examined via confocal microscopy. Representative images and the analysis are shown. All the data are presented as the means±SDs of at least three independent experiments. *p<0.05, **p<0.01, ***p<0.001.

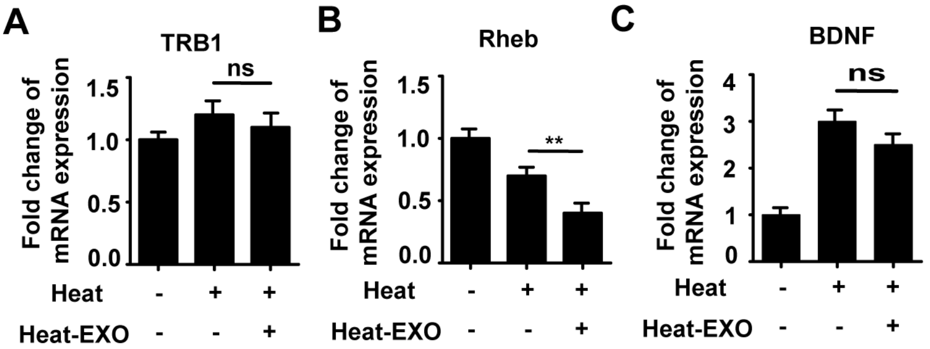

Figure S4: Screening the target of miR-155 in neuron. N2a cells cultured alone or incubated with exosomes derived from heat-stressed microglia for 24 h to allow enough exosomal uptake and then subjected to heat stress at 42°C for 2 h, followed by a 6 h recovery period at 37°C, the expression of TRB1 **(A)**, Rheb **(B)** and BDNF **(C)** in N2a cells was measured by qRT-PCR and normalized to HPRT expression. All the data are presented as the means±SDs of at least three independent experiments. **p<0.01.

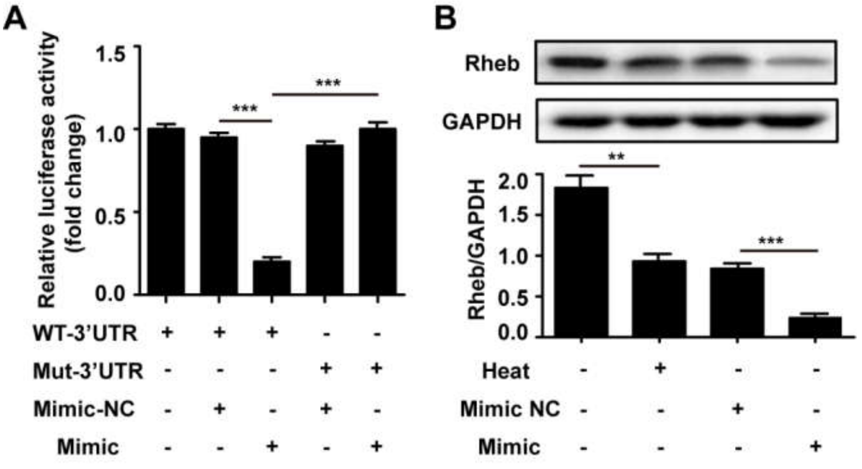

Figure S5: Rheb is the target of miR-155. (A) The luciferase reporter plasmids carrying the WT or Mut 3'UTR of Rheb were cotransfected with either the miR-155 mimic or miR-155 mimic-NC into 293T cells, after 24h, luciferase activity was detected. (C) N2a cells were transfected with miR-155 mimic or its negative control for 24h, and then subjected to heat exposure at 42°C for 2h, followed by a 6h recovery period at 37°C. The protein expression of Rheb in N2a cells was determined by Western bloting. Densitometric analysis was performed. All the data are presented as the means±SDs of at least three independent experiments. **p<0.01, ***p<0.001.

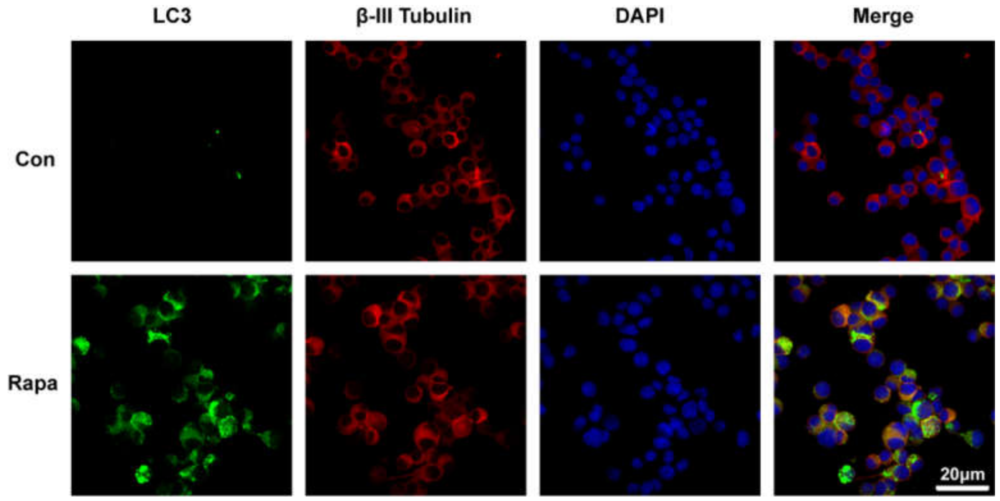

Figure S6: The effects of Rapa on neuronal autophagy. N2a cells were treated with or without 100 nM Rapa, the immunolocalization of LC3II and β-III Tubulin in the cytoplasm of N2a cells was examined via confocal microscopy.
